# The persistence and evolutionary consequences of vestigial behaviours

**DOI:** 10.1111/brv.12847

**Published:** 2022-02-26

**Authors:** Jack G. Rayner, Samantha L. Sturiale, Nathan W. Bailey

**Affiliations:** ^1^ Centre for Biological Diversity, Harold Mitchell Building University of St Andrews St Andrews KY16 9TH U.K.; ^2^ Present address: Department of Biology Georgetown University 3700 O St NW Washington DC 20057 U.S.A.

**Keywords:** non‐adaptive behaviour, preadaptation, relaxed selection, trait loss, trait reversal, vestigial trait

## Abstract

Behavioural traits are often noted to persist after relaxation or removal of associated selection pressure, whereas it has been observed that morphological traits under similar conditions appear to decay more rapidly. Despite this, persistent non‐adaptive, ‘vestigial’ behavioural variation has received little research scrutiny. Here we review published examples of vestigial behavioural traits, highlighting their surprising prevalence, and argue that their further study can reveal insights about the widely debated role of behaviour in evolution. Some vestigial behaviours incur fitness costs, so may act as a drag on adaptive evolution when that adaptation occurs *via* trait loss or reversal. In other cases, vestigial behaviours can contribute to future evolutionary trajectories, for example by preserving genetic and phenotypic variation which is later co‐opted by selection during adaptive evolution or diversification, or through re‐emergence after ancestral selection pressures are restored. We explore why vestigial behaviours appear prone to persistence. Behavioural lag may be a general phenomenon arising from relatively high levels of non‐genetic variation in behavioural expression, and pleiotropic constraint. Long‐term persistence of non‐adaptive behavioural traits could also result when their expression is associated with morphological features which might be more rapidly lost or reduced. We propose that vestigial behaviours could provide a substrate for co‐option by novel selective forces, and advocate further study of the fate of behavioural traits following relaxed and reversed selection. Vestigial behaviours have been relatively well studied in the context of antipredator behaviours, but they are far from restricted to this ecological context, and so deserve broader consideration. They also have practical importance, with mixed evidence, for example, as to whether predator/parasite‐avoidance behaviours are rapidly lost in wildlife refuges and captivity. We identify important areas for future research to help determine whether vestigial behaviours essentially represent a form of evolutionary lag, or whether they have more meaningful evolutionary consequences distinct from those of other vestigial and behavioural traits.

## I. INTRODUCTION

While much evolutionary research addresses the origins of novel traits or elaboration of existing traits, trait loss or reduction contributes prominently to observed patterns of evolutionary change (Fong, Kane & Culver, 1995; Wiens, 2001; Porter & Crandall, 2003; Lahti *et al*., 2009), and is an important prediction of major theoretical models in evolutionary biology (Fisher, 1958; Lande, 1981). Trait reversals have been widely studied, having attracted renewed attention in recent decades, and provide some of the most compelling and widely appreciated examples of evolutionary adaptation (McNab, 1994; Jeffery, 2005; Zuk, Rotenberry & Tinghitella, 2006; Xie *et al*., 2019). However, the bulk of this research has concerned the loss or reduction of morphological or physiological traits. With notable exceptions, the fate of behavioural traits following relaxed selection has been studied less often, probably due to the difficulty of identifying quantitative changes in behaviour without detailed first‐hand observation (Messler *et al*., 2007). On the contrary, behavioural traits are often assumed to be highly flexible and adaptable, or labile in their expression (Schmalhausen, 1949; Gomulkiewicz & Kirkpatrick, 1992), affording animals the ability to respond rapidly to changes in environment.

Vestigial behaviours have, however, been well characterised in a few amenable systems, and authors have repeatedly observed that behavioural trait loss appears to lag behind that of associated non‐behavioural traits under relaxed selection (Magurran *et al*., 1995; Coss, 1999; Plath *et al*., 2008; Lahti *et al*., 2009; Wund *et al*., 2015; Schneider *et al*., 2018; Rayner *et al*., 2019), such that behaviours often remain expressed after the selection pressures which once favoured them are removed or attenuated. For example, village weavers (*Ploceus cucullatus*) introduced from the African continent to islands of Hispaniola and Mauritius retain foreign egg‐rejection behaviours which are beneficial in resisting the cuckoo brood parasites present in their native range, but absent in their new habitats (Lahti, 2006). Yet, their ability to express this behavioural defence has been undermined by the concurrent loss of a complementary morphological trait: distinctive egg shell pigmentation, which in their ancestral habitat serves to help them discriminate between their own eggs and those of the brood parasite (Lahti, 2005). This pattern of ‘behavioural lag’ has parallels in a well‐characterised predator–prey system: the long‐term persistence of anti‐snake behaviours in Californian ground squirrel (*Spermophilus beecheyi*) populations which have evolved without the snake predators that were in their ancestral range (Coss, 1999). While many behavioural defences remain, physiological resistance to snake venom has been rapidly attenuated (Coss, 1999).

How common are non‐adaptive vestigial behaviours, and what are their implications for understanding the role of behaviour in evolution? We find they are prevalent in the literature, and propose that vestigial behaviours have the potential to influence contemporary and future evolutionary and ecological population dynamics. Vestigial behaviours could be influential, for example, if they are co‐opted for other adaptive functions (West‐Eberhard, 2003), or if their ability to persist largely or completely unexpressed preserves cryptic genetic variation. They also warrant consideration in behavioural research. Vestigial behaviours might re‐emerge under artificial experimental conditions, and may be prone to misinterpretation by researchers seeking to understand behavioural variation within an adaptive context (Gould & Lewontin, 1979; Dalos *et al*., 2021). For example, Byers (1997) controversially argued that many behavioural and morphological adaptations of American pronghorn (*Antilocapra americana*) populations are in fact adaptations to so‐called ‘ghosts of predators past’, that is, predators that have been long extinct (see also Peckarsky & Penton, 1988). Another illustrative example occurs in the Californian ground squirrels described above, among which individuals from populations that are not threatened by predatory snakes nevertheless show caution approaching sticks with a superficial snake resemblance (Coss, 1999). This fear of sticks might appear a peculiar trait to an observer unaware that snakes exert selection pressures in the squirrels' ancestral range.

## II. VESTIGIAL BEHAVIOURS AND MORPHOLOGY‐LED TRAIT LOSS

### (1) What is a vestigial behaviour?

We consider a *vestigial behaviour* to be any behavioural trait that is, or can be, expressed to some degree and which was once maintained by selection but has become *non‐adaptive* under the contemporary selection regime. Vestigial behaviours represent a category of non‐adaptive behaviours, which can arise through a variety of other routes (Bailey, 2013). Vestigial traits are often defined with the condition not just of having lost adaptive value, but also being evolutionarily atrophied, diminished, or reduced in expression (Fong *et al*., 1995). However, there is disagreement about whether trait reduction is a necessary condition for a trait's designation as vestigial, with others arguing that reduction is a likely consequence rather than a defining feature of traits being expressed in a vestigial state (Griffiths, 1993). As will be explored below, the likelihood, rate, and extent of evolutionary reduction in expression may depend on the type of trait involved and be qualitatively different for behaviours as a result of their typically flexible expression. So that we may consider whether and how vestigial behaviours are unique in their evolutionary origins, manifestation, and consequences, we adopt an encompassing definition that does not require expression of a behaviour to be quantitatively reduced (Griffiths, 1993). Thus, we consider the ‘vestige’ to be that which remains of a trait after the selection pressure maintaining it is relaxed or reversed. Reduced expression may nevertheless occur for vestigial behavioural traits through genetic or plastic mechanisms (Coss, 1999; Mooring *et al*., 2006), but such reduction may be more difficult to observe compared with that of non‐behavioural traits. Table 1 provides a glossary of the key terms used in this review.

**Table 1:** Glossary of terms

Non‐adaptive trait	A trait that does not confer a net fitness benefit to the organism expressing it. Its expression may be costly (maladaptive) or neutral.
Preadaptation	Potential for a trait to acquire a new adaptive function, if changed selection favours a different function.
Relaxed selection	A state in which a selection pressure has been alleviated, so that the associated trait is no longer under direct selection.
Reversed selection	A state in which a previous selection pressure has been reversed rather than simply weakened. For example, if selection previously favoured the maintenance or elaboration of a trait, then after selective reversal the trait will be under negative selection.
Vestigial behaviour	A behavioural trait that was previously adaptive but has been rendered non‐adaptive by relaxed or reversed selection. Its expression might have been quantitatively reduced, for example if expression is context dependent or has been partly undermined by genetic changes, or it might remain expressed at similar levels.
Vestigial trait	Any trait that was previously adaptive but is non‐adaptive in the contemporary selective regime. The trait might be behavioural (see above) or non‐behavioural: including morphological, physiological, and life‐history traits. Such traits may also be described as ‘relicts’, ‘obsolete’ or ‘non‐functional’.

### (2) Relaxed and reversed selection

Reduction or loss of a behaviour is more likely under reversed selection than under relaxed selection. Assuming that a trait has a heritable genetic basis, if the selection pressure that previously favoured the trait is reversed then the reduction or loss of the associated trait would be expected to proceed due to negative selection (Hall & Colegrave, 2008). If selection is relaxed but not reversed, as for traits that no longer exert fitness benefits but are not themselves costly to produce or maintain, the non‐functional trait might persist over longer evolutionary timescales. The same is true of behavioural traits that remain unexpressed or are shielded from selection by the absence of morphological appendages necessary to express the behaviour. For traits under relaxed selection, i.e. that are or are nearly selectively neutral, accumulation of neutral mutations might slowly erode their expression (Haldane, 1933; Hall & Colegrave, 2008), allowing the trait to persist in vestigial form over many generations. These differences in exposure to selection under relaxed and reversed forms of selection can be observed in arthropod species that have made the transition to asexuality, among which there appears to be greater reduction of female *versus* male sexual traits (van der Kooi & Schwander, 2014). In asexual species, such traits are more likely to be costly to females but selectively neutral for males, due either to the rarity of males or to the fact that they do not contribute offspring (van der Kooi & Schwander, 2014). In many cases, it is likely that a combination of the above processes will occur: the non‐adaptive trait might be under negative selection until its expression is reduced such that selection is not sufficiently strong to favour further reduction (Lahti *et al*., 2009).

### (3) Why might behaviours persist longer than other vestigial traits?

**Fig. 1:**
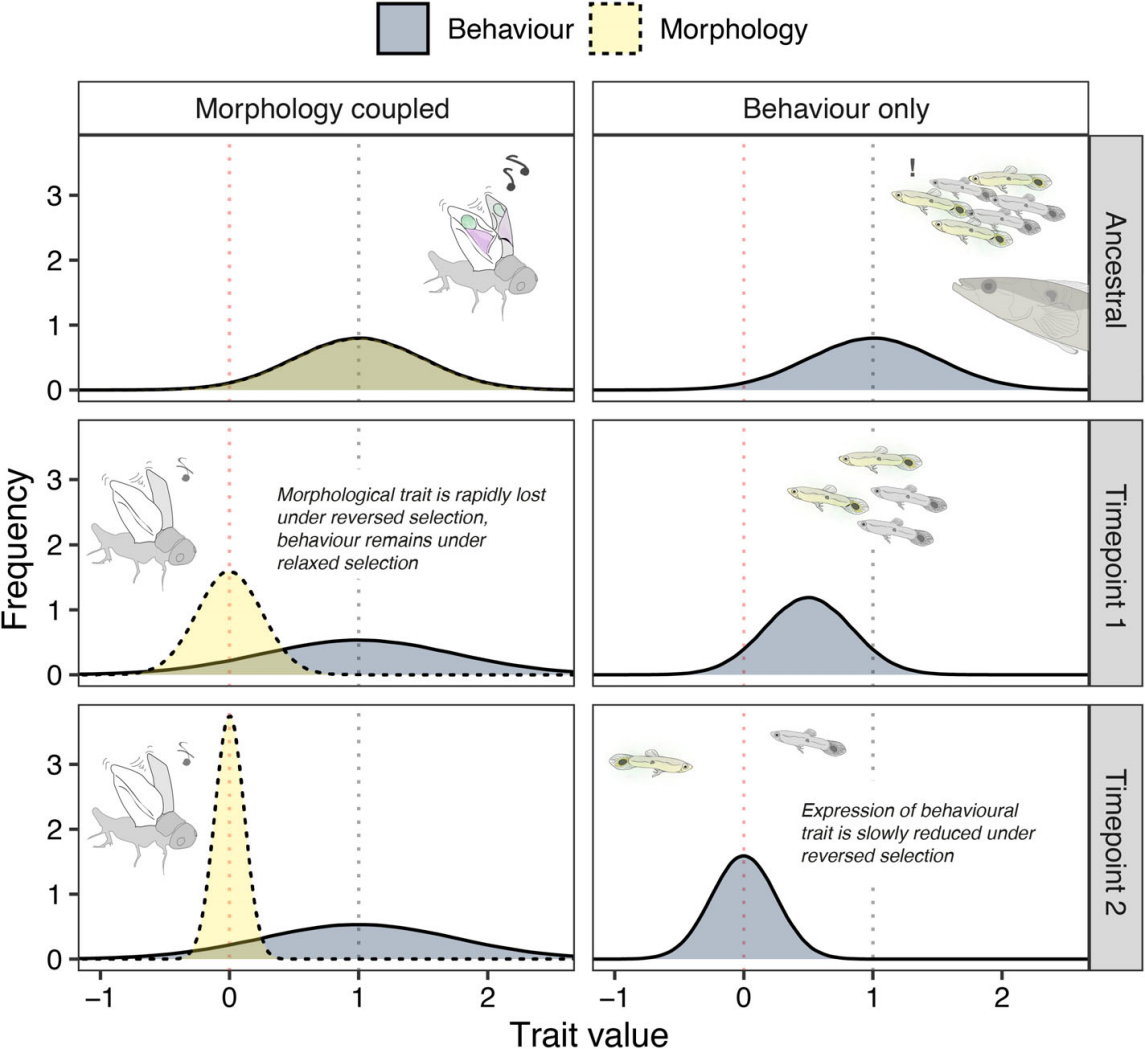
A vestigial behavioural trait is more likely to be retained if its expression involves a morphological trait that is more rapidly lost. The schematic illustrates the transition of a hypothetical population from an ancestral state, in which a trait variant increases fitness, to two timepoints following reversal of selection on that trait. Timepoint 1 represents a stage shortly after selection is reversed, showing rapid evolutionary dynamics (*ca*. 10–50 generations), whereas timepoint 2 represents a longer interval (*ca*. 100–1000 generations). Dotted lines at *x* = 1 and *x* = 0 represent fitness optima for the combined trait before and after selective reversal, respectively. The left panel represents a scenario in which the trait under negative selection is a combination of behavioural (blue) and morphological (yellow) components [such as in the illustrated example of song‐loss in Hawaiian oceanic field crickets *Teleogryllus oceanicus*, where behavioural singing effort remains despite rapid genetic loss of sound‐producing structures on male forewings due to eavesdropping parasitoids (see Table 2)]. In the right panel, behavioural expression is itself under reversed selection [as in the non‐adaptive schooling behaviour in populations of guppies *Poecilia reticulata* in low‐predation environments (Table 2)]. In the former example, only one component of the trait need be lost for the trait to be non‐functional, and existing evidence suggests that morphological traits are typically more rapidly attenuated, leaving the associated behavioural trait to persist under relaxed selection. In the absence of morphological trait loss, behavioural expression may be gradually reduced under selection. In both scenarios we assume that negative selection reduces phenotypic variance of the relevant trait.

Behavioural traits have been repeatedly observed to persist for longer under relaxed selection than other traits (Fong *et al*., 1995; Lahti *et al*., 2009; Wund *et al*., 2015). This pattern has not been subject to quantitative analysis, but this surprising observation nevertheless warrants consideration. Assuming they are genetically variable, non‐adaptive behaviours should be subject to the same forces that drive the evolutionary reduction of, for example, morphological traits. What then could explain the apparent persistence of non‐adaptive behaviours compared with non‐behavioural traits? First, if the loss of morphological or physiological traits does typically offer a path of least resistance for adaptive trait reduction, then this could have the direct effect of shielding associated behaviours from selection (Fig. 1). For example, socially parasitic ant species which rely on brood care and social infrastructure provided by closely related host species have lost genes associated with olfactory receptors (Schrader *et al*., 2021), which underpin chemical communication and are an essential component of sensory input that stimulates expression of social behaviour (Trible *et al*., 2017). Thus, selection can reduce expression of a behaviour by favouring loss of sensory inputs required to activate the behaviour, rather than loss of components of the behaviour itself.

But what, in the first place, could explain the apparent persistence of non‐adaptive behaviours compared with non‐behavioural traits during the early stages of trait reduction? Heritability of behaviour is typically low compared with morphology (Mousseau & Roff, 1987; Stirling, Réale & Roff, 2002). This could be due in part to selection eroding genetic variation underlying fitness‐associated behavioural traits. However, incorporating measures of behavioural repeatability tends to increase heritability estimates (Dochtermann, Schwab & Sih, 2015) suggesting low observed heritability may be partly due to the difficulty of reliably measuring behavioural traits. This follows from the fact that behavioural expression shows considerable intra‐individual variation (Bell, Hankison & Laskowski, 2009), being typically flexible or context specific, and strongly influenced by factors such as social experience (Bailey, Gray & Zuk, 2010) and abiotic environment (Graf & Sokolowski, 1989). Note, however, that even flexibly expressed behaviours are not necessarily endlessly plastic, or labile in their expression; while behaviours themselves are nearly always ‘reversibly’ expressed, reaction norms and behavioural phenotypes may be experience‐based (Blumstein, 2002), or less malleable outside crucial developmental periods (Duckworth, 2009). Nevertheless, the context‐dependent expression of behavioural traits means that they might often persist under relaxed selection, exerting little to no cost, whereas non‐behavioural traits are likely to incur fitness costs associated with their development, maintenance, and constitutive expression. Consequently, the heritability of and selection on behavioural traits, each necessary for evolutionary responses to occur (Price, 1970; Falconer, 1981), are complicated by high levels of non‐genetic variation in their expression.

Behavioural traits are also frequently correlated within and across ecological contexts (Sih, Bell & Johnson, 2004), which is likely to increase the level of evolutionary constraint to which individual behaviours are subject (Dochtermann & Dingemanse, 2013; Royauté, Hedrick & Dochtermann, 2020) and further hinder their adaptive loss. In some instances this integration may be the result of genetic covariance, perhaps arising from correlated selection on ecologically related traits, in others it might simply arise from shared neural architectures underpinning various behaviours and physiological processes, including those that are constitutively expressed (Tierney, 1996). There are, therefore, a number of reasons to anticipate that behavioural trait loss might be subject to distinctive evolutionary dynamics under relaxed or reversed selection, in effect delaying or stymieing their evolutionary reduction. The extent to which each of these features explains the maintenance of vestigial behaviours is however unclear and will vary across traits.

## III. EXAMPLES OF VESTIGIAL BEHAVIOURS ACROSS CONTEXTS

**Fig. 2:**
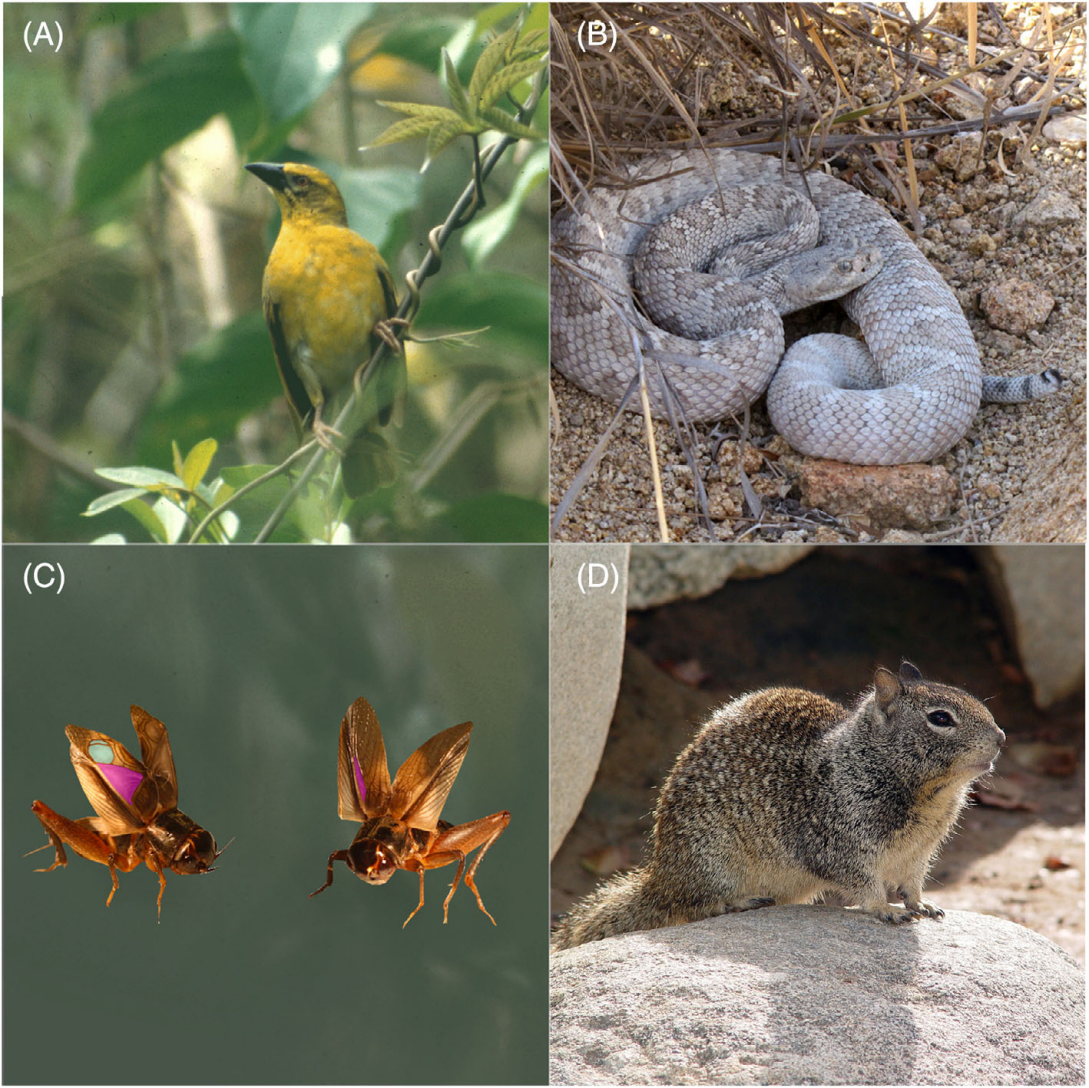
Species reported to exhibit behaviours which, due to changes in ecology or morphology, are rendered non‐adaptive. (A) Village weavers *Ploceus cucullatus* retain foreign egg‐rejection behaviours hundreds of years after colonising islands on which brood parasitism was (until recently) absent. However, their ability to discern foreign eggs has been undermined by morphological changes in egg appearance under this relaxed selection (Lahti, 2006). Photograph credit: David Lahti. (B) The rattlesnake *Crotalus catalinensis* has through morphological change lost the ability to produce the characteristic ‘rattling’ signal, likely due to the absence of larger predators. Nevertheless, this species still exhibits ‘rattling’ behaviour by shaking its tail when threatened (Shaw, 1964). Photograph credit: Gustavo Arnaud. (C) In Hawaiian populations of oceanic field crickets *Teleogryllus oceanicus*, males continue to express energetically costly wing movements associated with the production of song, despite the loss or reduction of morphological structures on their wings which renders them silent (compare highlighted wing features in the normal‐wing male, left, with those of the silent ‘flatwing’ male, right) (Schneider *et al*., 2018). Original cricket photograph credits: Nathan Bailey. (D) Ground squirrels *Otospermophilus beecheyi* retain the ability to recognise and express anti‐predator behaviours targeted to predatory snakes present in their ancestral range up to 300,000 years after colonising habitats in which the snakes are absent, whereas resistance to snake venom was attenuated more rapidly (Coss, 1999). Photograph credit: Howard Cheng, made available under CC BY‐SA 3.0 (https://creativecommons.org/licenses/by‐sa/3.0/). In these four examples, non‐behavioural (morphological or physiological) traits were lost or reduced, whereas associated non‐adaptive behavioural traits remained.

Vestigial behaviours have been observed to persist after the loss of associated function or selection pressure across a range of contexts. Selected examples are summarised in Table 2 and Fig. 2, and the ecological contexts surrounding some of these examples of non‐adaptive behavioural persistence are discussed below. We focus on anti‐predator/parasite defence and signalling behaviours, in which much of the work looking at vestigial behaviours has been conducted due to the relative ease of inferring changes in selection regime (Coss, 1999; Blumstein, 2006; Lahti, 2006; Peer *et al*., 2011). We note however that these are likely to be far from the only ecological contexts in which vestigial behaviours persist.

**Table 2:** Selected examples of vestigial behaviours across ecological contexts

Ecological context	Species	Behavioural trait	Change in selection	Timescale	Notes	Evolutionary reduction of associated non‐behavioural traits?^a^	References
Anti‐predator behaviour	Village weavers (*Ploceus cucullatus*)	Egg‐rejection behaviour	Absence of cuckoo brood parasite	From 18th century until introduction of cowbird brood parasites in the 1970s. In other species, vestigial egg‐rejection behaviours have persisted for up to 3 million years (Peer *et al*., 2011).	Behavioural egg rejection is undermined by morphological loss of egg pigmentation.	Yes – loss of distinctive egg pigmentation	Lahti (2005, 2006)
	California ground squirrel (*Spermophilus beecheyi*)	Anti‐predator behaviours in response to snakes	Absence of predatory snakes	Variable, but all behaviours persisted to some degree for 70,000 to 300,000 years after selection was relaxed	Some anti‐snake behaviours appear changed in populations under relaxed selection, others are not. Venom resistance has been reduced in populations under relaxed selection.	Yes – reduced venom resistance	Coss (1999)
	Trinidadian guppies (*Poecilia reticulata*)	Predator defence schooling behaviours	Experimental relocation to low‐predation habitat	16 years (*ca*. 50 generations)	The low‐predator population rapidly evolved greater male conspicuousness, while offspring and female reproductive masses also shifted, but schooling behaviour appeared unchanged.	Yes – brighter male colouration and changes to multiple life‐history traits	Reznick, Bryga & Endler (1990); Magurran *et al*. (1995)
	Threespine stickleback (*Gasterosteus aculeatus*)	Multicontextual antipredator responses	Relaxation of predation threat due to colonisation of freshwater lakes	Up to 20,000 years	Evidence of rapid elaboration of antipredator responses after secondary introduction of trout predators, suggesting vestigial anti‐predator behaviours might have played an important role in facilitating subsequent resistance to a novel predator.	Yes – substantial armour reduction in derived freshwater populations	Messler *et al*. (2007); Wund *et al*. (2015)
	Baltic clams (*Macoma balthica*)	Evasive burrowing behaviour	Geographic expansion to a habitat where crab predators are absent	Several thousand years, inferred from glacial movement	Populations that are or are not exposed to predatory crabs in their natural range show quantitatively similar burrowing responses to experimental crab exposure.	–	Edelaar, Piersma & Postma (2005)
	Impala (*Aepyceros melampus*), blue wildebeest (*Connochaetes taurinus*), and warthogs (*Phacochoerus africanus*)	Response to calls of native African lion (*Panthera leo*) predator	Absence of predator stimuli	Up to *ca*. 20 generations at the time of study	Naïve and lion‐exposed populations showed quantitatively similar expression of antipredator behaviours in response to playback of lion calls.	–	Dalerum & Belton (2015)
Anti‐parasite behaviour	Bighorn sheep (*Ovis canadensis mexicana*)	Anti‐tick grooming behaviours	Absence of ticks in a desert population	Unknown, but ticks are suggested to have been absent for hundreds or thousands of years	Although the population still exhibited grooming behaviour, it did so at a relatively low level compared to other ungulates. Grooming behaviour was negatively associated with body size, consistent with expectations that in the presence of ticks, smaller animals should pay a higher cost of ectoparasitism.	–	Mooring *et al*. (2006)
Anti‐predator/ warning signal	Santa Catalina rattlesnake (*Crotalus catalinensis*)	Conspicuous tail ‘rattle’ warning signal	Absence of large ungulates and predators	Unknown, but divergence of C. *catalinensis* from *C. ruber* occurred *ca*. 3.67 million years ago	Shaw (1964) and others reported that rattlesnakes which have only a vestigial ‘rattle’, nevertheless readily perform silent rattling behaviour by twitching their tails when threatened by approaching humans.	Yes – reduction of rattle structures	Shaw (1964); Radcliffe & Maslin (1975); Arnaud & Martins (2019); Ruiz‐Sanchez *et al*. (2019)
Courtship/mating	Several (e.g. *Drosophila*, *Phasmida* stick insects, several species of parasitoid wasp)	Courtship and mating behaviour	Transition to asexuality	Variable	Male courtship and mating behaviours (evolutionarily neutral due to males having negligible fitness) are more often retained in asexual species compared with those of reproducing females, which are likely to involve fitness costs through, e.g. metabolic expenditure.	Mixed – frequently yes, with mating behaviours often persisting in the absence of necessary morphology. Behavioural decay when it does occur is nearly always accompanied by morphological/physiological trait loss.	van der Kooi & Schwander (2014) and references therein
Sexual signalling behaviour	Oceanic field crickets (*Teleogryllus oceanicus*)	Wing movements that ordinarily generate acoustic signals	Morphological loss of ability to sing	>50–70 generations	At least five genetically distinct silent male morphs still exhibit wing motor patterns associated with singing. Some show no reduction in energetically costly singing effort after *ca*. 50 generations of obligate silence.	Yes – loss or reduction of sound‐producing features on wings	Schneider *et al*. (2018); Rayner, Schneider & Bailey (2020)
Signal receptivity	Atlantic molly (*Poecilia mexicana*)	Visual‐cue‐based mating interactions (audience effects, mate choice)	Complete darkness	Cave‐dwelling and surface populations are suggested to have diverged up to 10,000 years ago (McGowan *et al*., 2019)	Cave‐dwelling populations also appear to have evolved perception of non‐visual size cues in evaluating conspecifics, perhaps using the ‘lateral line system’ of tactile sensory organs.	Yes – eye structures are reduced, although remain functional (contrasting with other cave fishes).	Plath *et al*. (2004, 2008)

^a^ Dashes indicate no information.

### (1) Anti‐predator and anti‐parasite behaviours

Predator–prey (or host–parasite) dynamics lend themselves to empirical study of vestigial behaviour because relaxed selection can be inferred by the absence of key predator or parasite species. Populations often express specialised defence behaviours that are advantageous primarily under selection conferred by a single species or group of species in their range (Peer *et al*., 2011). If a prey species colonises a new habitat where the associated predator species is absent, or predators go locally extinct, then it might be expected that associated anti‐predator behaviours will be lost in the manner frequently observed among morphological defence or predator‐avoidance traits (Xie *et al*., 2019). Yet evidence for this is mixed, and in many cases anti‐predator behaviours persist long after selection is relaxed.

In Section I we discussed the persistence of anti‐snake behaviours among Californian ground squirrel populations in habitats where snakes are absent. While the extent to which such behaviours persist varies, some defences such as substrate‐throwing and the ability to distinguish between snake species are observed in populations hundreds of thousands of years after selection was relaxed, whereas physiological resistance to snake venom evolved much more rapidly (Coss, 1999). We also mentioned vestigial anti‐predator/parasite behaviour expressed by village weavers which continued to exhibit egg‐rejection behaviours for at least 100–200 years after their colonisation of Hispaniola, an island on which cuckoo parasites native to their ancestral range are absent (Lahti, 2006). Again, morphological trait loss proceeded *via* loss of distinctive egg pigmentation which facilitates species discrimination (Lahti, 2005), while behavioural defences remained intact. Intriguingly, one of the reasons it is difficult to determine how long this vestigial behaviour remained intact under relaxed selection is that, at some point in the late 20th century, shiny cowbird (*Molothrus bonariensis*) brood parasites were introduced to Hispaniola (Lahti, 2006). Persistence of the vestigial egg‐rejection behaviour could therefore have contributed to this population's resistance to a novel evolutionary threat – although Lahti (2006) notes that, unlike the cuckoos in their native range, the distinctive appearance of the shiny cowbird's eggs may not require the maintenance of specific egg‐recognition behaviours. Consistent evidence of persistent behavioural defences against nest brood parasitism *via* egg‐rejection behaviour has been reported across a range of species (Rothstein, 2001), with egg‐rejection behaviours apparently persisting for up to 3 million years under relaxed selection (Peer *et al*., 2011).

Messler *et al*. (2007) studied whether the well‐documented loss of morphological defences against predators in threespine stickleback (*Gasterosteus aculeatus*) freshwater populations, which typically experience lower predator abundance, had been accompanied by changes in behavioural responses. They compared predator defence behaviours in populations from contrasting predator regimes: an oceanic population representative of an ancestral high‐predation environment; a freshwater population devoid of predators representative of typical low‐predator derived freshwater environments; and a second derived freshwater population occupying a lake into which predatory trout (*Oncorynchus mykiss*) had been recently introduced. They found little evidence of a general reduction in anti‐predator behaviours in the freshwater populations compared with the ‘ancestral’ population, although there was some indication of heightened responsiveness to trout in the freshwater population secondarily exposed to predation. Wund *et al*. (2015) further found that this secondary exposure to trout predators selected for antipredator responses, but that ancestral and trout‐naïve freshwater populations showed few or no differences in antipredator responses to begin with. Anti‐predator behaviours retained under relaxed selection may therefore increase evolutionary capacity for resistance against future predator threats. This consequence of a vestigial behaviour can be contrasted, for example, with the recurrent reduction of morphological defensive armour in freshwater threespine stickleback populations, which in at least one case contributed to the population's extinction after predatory pike (*Esox lucius*) were introduced (Patankar, Von Hippel & Bell, 2006).

The persistence of non‐adaptive anti‐predator behaviours can also interfere with other fitness‐associated traits, in which case they should be under reversed selection. Schooling behaviour, for example, while an effective anti‐predator defence, interferes with intra‐specific interactions by reducing the ability of individuals to defend resource patches (Huntingford, 1982; Seghers & Magurran, 1991), and is predicted to be under reversed selection in low‐predation environments (Seghers & Magurran, 1994). Yet, in Trinidadian guppies (*Poecilia reticulata*), initial laboratory‐based behavioural assays found no evidence that populations transplanted from a high‐predation environment to a low‐predation environment evolved reduced anti‐predator behaviour in the short term, despite concurrent changes in life‐history traits and the elaboration of conspicuous male colouration (Reznick & Endler, 1982; Reznick *et al*., 1990; Magurran *et al*., 1995). Magurran *et al*. (1995) described as puzzling their finding that behavioural traits lagged behind the evolution of morphological and life‐history traits (e.g. Reznick *et al*., 1990) in responding to changes in predator abundance, but noted evidence from another population in which antipredator behaviours were eventually reduced, although over timescales approximately twice as long (*ca*. 100 generations) (see Magurran *et al*., 1992).

Like anti‐predator behaviours, parasite‐defence behaviours are predicted to be under relaxed selection when populations are no longer exposed to parasites. However, Mooring *et al*. (2006) found that tick‐defence grooming behaviours have persisted in a desert population of bighorn sheep (*Ovis canadensis mexicana*), albeit at a relatively low rate compared with other ungulates of a similar size, despite ticks apparently having been absent for thousands of years. Similarly, Li, Beauchamp & Mooring (2014) found that populations of Père David's deer (*Elaphurus davidianus*) which had been bred in captivity over very long intervals (*ca*. 800 years), nevertheless generally retained patterns of grooming behaviours which are adaptive in the presence of ticks, despite their supposed absence or near‐absence in the captive environment. A factor that may be of importance to this example of behavioural persistence under relaxed selection is that the captive deer population was subject to an extreme bottleneck (with the world's population of *E. davidianus* descending from just 18 captive individuals) (Li *et al*., 2014), reducing heritable genetic variation which may have impeded the behaviour's loss regardless of the relaxation of selection.

While vestigial anti‐predator behaviours appear relatively common, there are also examples in which such behaviours have been lost, sometimes rapidly, under relaxed or reversed selection (Stoks, McPeek & Mitchell, 2003; Stankowich & Coss, 2007; Fowler *et al*., 2018; Jolly, Webb & Phillips, 2018). This has important consequences for conservation biology, as population management schemes that remove predator threat could lead to the loss of traits such as neophobia, negatively impacting fitness if populations are again exposed to predators (Muralidhar *et al*., 2019; Geffroy *et al*., 2020; Jolly & Phillips, 2021). When a population has been maintained for many generations in an artificial environment such as a zoo or wildlife reserve that is largely or completely predator/parasite‐free, it is important to consider whether anti‐predator or anti‐parasite behaviours have been lost before initiating its reintroduction into the wild (Wei *et al*., 2013; Li *et al*., 2014). Similarly, a recent impetus to reintroduce large terrestrial carnivores to parts of their ancestral range in which they have long been absent has led to concern that prey species in these areas may have lost their predator recognition and defensive behaviours. Study of behavioural loss or retention in this context is necessary to predict whether prey species are likely to experience predator‐driven population declines, or whether (and under what conditions) vestigial anti‐predator behaviours might facilitate survival upon reintroduction of natural enemies (Berger, Swenson & Persson, 2001; Muralidhar *et al*., 2019; Geffroy *et al*., 2020; Jolly & Phillips, 2021).

### (2) Signalling behaviour

The bulk of research into vestigial behaviours has focussed on predator–prey interactions, with various hypotheses having been proposed to explain their persistence under relaxed selection, including multicontextuality of antipredator vigilance behaviours, context‐dependent expression and pleiotropic constraint (Peckarsky & Penton, 1988; Coss, 1999; Blumstein, 2006). Yet there is no reason to expect that vestigial behaviours should be restricted to this ecological context. Signals function in intra‐ and inter‐specific displays, such as mate attraction and predator avoidance, but also typically involve fitness costs. For example, sexually selected signals that attract mates also frequently attract the attention of predators or other unintended receivers (Zuk & Kolluru, 1998). Signals also tend to involve energetic costs associated with their development and/or expression. If these costs come to outweigh the benefits of the signal then it will be under negative selection. Consistent with this, conspicuous sexual signals are frequently lost (Wiens, 2001). The fate of associated behaviours is less well understood and has not often been studied but, like anti‐predator behaviours, lends itself to empirical study given the relative ease of inferring relaxed or reversed selection following the loss of the associated signal. This is an important gap in our understanding of sexual signals, as the retention of signalling behaviour components could play a vital role in facilitating their re‐emergence after evolutionary reversal (Broder *et al*., 2021a).

Recent work has addressed the fate of behavioural traits underlying a sexually selected signal, male song, in Hawaiian oceanic field crickets, *Teleogryllus oceanicus*. Populations on multiple islands are parasitised by the offspring of an acoustically orienting parasitoid fly, which uses male cricket song to locate hosts. Under this selection pressure, males in populations on multiple islands have adaptively lost the ability to sing due to changes in forewing morphology, which prevents the wings from producing sound when males elevate them and rub them together (Fig. 2C). Loss of song through morphological evolution has occurred through reduction of male‐specific patterns of wing venation required to produce sound (the ‘flatwing’ phenotype), on at least three separate occasions through independent genetic mutations (Zuk *et al*., 2006; Pascoal *et al*., 2014; Zhang *et al*., 2021). In addition, song has also been lost through changes in wing size (‘small‐wing’) and changes in three‐dimensional wing configuration (‘curlywing’) which each preclude proper engagement of sound‐producing features and thereby result in protective male silence (Rayner *et al*., 2019). Intriguingly, song loss has not to our knowledge evolved *via* reduction or cessation of behaviours associated with calling, i.e. behavioural reduction. Instead, all of the silent morphotypes still move their forewings in a stereotyped motor pattern typical of calling, despite the fact that this movement fails to produce the associated acoustic signal.

In the case of flatwing phenotypes, it is also known that singing effort persists unchanged in silent and predominantly silent wild populations for at least 50 generations following the initial appearance of silent morphotypes (Rayner *et al*., 2020). This example of parallel behavioural persistence is surprising given the energetic costs with which song production is associated (Cade, 1991; Hack, 1998), suggesting it should be subject to reversed selection following the loss of song, and because the behaviour is consistently or even constitutively expressed. However, song‐associated wing movements in crickets are induced by central pattern generators (Schöneich & Hedwig, 2011). These networks of neurons are highly integrated across behaviours, and thus tend to be evolutionarily conserved, as mutations affecting such networks would be highly likely to affect other important adaptive behaviours (Tierney, 1996). Mutations which disrupt the neural architecture underlying a cricket's behavioural ability to sing may therefore be strongly deleterious in other contexts. Interestingly, recent evidence suggests that this retention of singing motor behaviour in silent crickets could facilitate evolutionary re‐emergence of the sexual signal, perhaps with different acoustic properties (Schneider *et al*., 2018; Bailey, Pascoal & Montealegre, 2019). This might be expected in cases where benefits of mate attraction outweigh risks of attracting predators or parasites, or in which ‘clandestine’ communication is possible (Tinghitella *et al*., 2018, 2021; Broder *et al*., 2021b).

In the *Libellago* genus of damselflies, males of several species vibrate their white‐coloured legs as an excitatory signal during courtship. Males of two species, *L. hyalina* and *L. semiopaca*, do not exhibit white‐ornamented legs: the former does not employ leg vibration in courtship, whereas the latter does (Orr, 1995). It is not clear whether the leg‐vibration expressed by *L. semiopaca* represents an ancestral behaviour that facilitated subsequent and complementary elaboration of white leg ornamentation in other species, or rather a vestigial behaviour following the loss of leg ornamentation in this species. However, the latter scenario would be consistent with that outlined in Fig. 1, in which the loss of a morphological trait permits the persistence of associated non‐adaptive behaviours.

Examples of behavioural signalling persistence after relaxation of selection are not limited to sexual signals, but also include inter‐specific advertisement behaviours such as aposematic display. On the island of Santa Catalina, off the coast of Baja California, endemic rattlesnakes (*Crotalus catalinensis*) express only a vestigial rattle ornament (Fig. 2B), and so cannot produce the acoustic rattling warning signal characteristic of other rattlesnake species. It has been suggested that rattle morphology was lost under a combination of relaxed selection owing to the absence of large mammals that might disturb the snakes (Radcliffe & Maslin, 1975), and reversed selection for inconspicuousness which is beneficial in capturing prey (but see Avila‐Villegas, Martins & Arnaud, 2007). Despite loss of the acoustic signal, *C. catalinensis* continues to exhibit rattling behaviour by rapidly twitching its tail (Shaw, 1964; Allf, Durst & Pfennig, 2016). In related species, rattling behaviour is not highly energetically costly once the relative infrequency of the behaviour is taken into account (Moon, 2006), so it may not be subject to strong reversed selection in *C. catalinensis*. Nevertheless, the behaviour does involve energetic costs, and it is plausible the behaviour is expressed less frequently in silent populations, although time spent rattling does not appear to have been subject to quantitative analysis.

Loss of a signal will not only impact the behaviour of senders, but also that of receivers. A number of studies have found female mate preference to be mediated by the presence of predators, with females tending to exhibit a greater preference for larger or more elaborated males when predators are less abundant (Reznick *et al*., 1990; Godin & Briggs, 1996). However, the fate of such preferences following the loss of the associated signal or stimulus is less clear. In the case of sexual signals, their loss might have the effect of relaxing selection on the ability of receivers to perceive the associated signal through loss of acoustic or visual sensitivity (Fullard, Ratcliffe & Soutar, 2004), whereas behavioural changes may be less evident. Counter to this, females of a parthenogenetic katydid, *Poecilimon intermedius*, show little if any phonotaxis towards male song of a closely related species *P. ampliatus* (Lehmann *et al*., 2011), but retain acoustic sensitivity which likely serves other important ecological functions (Lehmann, Strauß & Lakes‐Harlan, 2007) suggesting an evolved reduction specifically in receptivity to male signals. By contrast, male cave‐dwelling Atlantic mollies (*Poecilia mexicana*) retain eye structures despite the absence of light in their cave habitats and continue to exhibit visual‐cue‐based mate choice when tested in a laboratory setting, indicating they retain receptivity to visual stimuli (Plath *et al*., 2004, 2008). However, females of cave‐dwelling populations have also evolved the ability to assess male size by non‐visual sensory cues (Plath *et al*., 2008). The evolution of behavioural responses to signals is, importantly, not restricted to reduction or persistence, but could involve changes in direction or strength of the response, which could become an important factor in any future re‐evolution of the associated signal. In silent Hawaiian oceanic field crickets, for example, females from populations evolving in the absence of song became more discerning in their evaluation of male calling song when song was restored by experimental playback, whereas the ancestral population showed the opposite response (Bailey & Zuk, 2012).

### (3) Other contexts in which vestigial behaviours have been observed

While changes in selection are readily inferred for anti‐predator/parasite and behavioural signalling behaviours, vestigial behaviours also persist in other ecological contexts. For example, van der Kooi & Schwander (2014) reviewed the fate of sexual traits following the transition to asexuality across arthropod taxa. They showed that many behaviours associated with courtship or mating remain intact long after selection is relaxed or removed entirely by the loss of sexual reproduction. Moreover, they demonstrate that non‐adaptive behaviours expressed by males, who if present at all are typically selectively neutral as they contribute few if any offspring, tend to persist for much longer than those expressed by females which remain under selection. Vestigial traits associated with sexual reproduction are likely to be maladaptive in females, so in general should be more rapidly attenuated under reversed selection (Hall & Colegrave, 2008). For example, laboratory experiments (Carson, Teramoto & Templeton, 1977; Carson, Chang & Lyttle, 1982) demonstrated rapid but heterogeneous reduction of female mating propensity in experimentally unisexual lines of *Drosophila mercatorum*.

Another context in which vestigial behaviours can be observed is the evolution of flightlesness in insects and birds (Roff, 1990; McNab, 1994). Among non‐flying stick insect, grasshopper and locust species, flight loss often involves the loss or reduction of wing structures (Roff, 1990; Whiting, Bradler & Maxwell, 2003), whereas functional circuitry underpinning the necessary wing movements is frequently retained (Kutsch & Kittmann, 1991; Kutsch, Martz & Gäde, 2002). Even wingless or brachypterous insect species frequently retain wing movement behaviours associated with flight (Kutsch & Kittmann, 1991; Viloria *et al*., 2003; Venn, 2007). Such behaviours can be readily observed when flight musculature is retained. However, neural circuitry underpinning flight‐associated wing movements can even be maintained in the absence of such musculature, which is energetically costly to develop and maintain (Roff, 1989; Katz, 2016). For example, grasshopper species incapable of flight because they lack the necessary musculature and appendages nevertheless retain much of the neuronal architecture that generates flight in related species (Arbas, 1983). Such conservation of neural mechanisms could have contributed to the repeated secondary evolution of flight in stick insects (Whiting *et al*., 2003). Similarly, flightless birds retain the ability to flap their wings (Katz, 2016), which could play an important facilitating role in the re‐emergence of flight were ancestral selection pressures to be re‐imposed.

Vestigial behaviours are frequently invoked as an alternative explanation for behaviours when an adaptive explanation is not forthcoming. Given the difficulty of reliably determining that a behaviour is vestigial (i.e. that it represents an ancestral state and no longer carries adaptive value), such assertions must be treated with caution, although they are useful to illustrate the potentially wide‐ranging evolutionary implications of vestigial behaviours. For example, brood parasites of many species have been observed to provision their own offspring even when their offspring are receiving parental care from the foster parents. Lorenzana & Sealy (1998) argue this is likely a vestigial brood‐provisioning behaviour, although they also consider (but consider less likely) that it could be an adaptive approach to supplementing provisioning provided by foster parents. Similarly, long‐distance migration of baleen whales between feeding and wintering grounds has been proposed to represent a vestigial behaviour owing to an ancestral state when smaller ocean basins meant that feeding and wintering grounds were nearer (Evans, 1987), whereas more recent evidence seems to indicate that migration is favoured by adaptive benefits associated with energy conservation (Rasmussen *et al*., 2007; Pitman *et al*., 2020). Further study of vestigial behaviours will be helpful in illuminating the circumstances and conditions under which such explanations of unexplained and seemingly non‐adaptive behaviours are justified.

## IV. FACTORS INFLUENCING THE PERSISTENCE AND LOSS OF BEHAVIOURAL TRAITS

### (1) Facultative expression and behavioural integration

What factors underlie the persistence of behaviours which are rendered non‐adaptive by a change in circumstances? As discussed above, a feature of particular relevance is that behaviours evolving under relaxed selection can persist at genetic and neural levels unexpressed, and therefore largely shielded from selection (Fong *et al*., 1995; Lahti *et al*., 2009). Even when expression of a behaviour *is* reduced or eliminated by selection, the underlying neurophysiology and therefore the ability to express that behaviour may be preserved (Katz, 2016; Gray *et al*., 2018). Table 3 provides general predictions regarding whether loss or reduction of behavioural traits is expected dependent on whether the trait is under relaxed or reversed selection, and whether expression of the trait is highly plastic or largely genetically influenced.

**Table 3:** Evolutionary predictions for vestigial traits. Each entry indicates the expected evolutionary change in trait value or expression under different scenarios, and for short‐term *versus* long‐term timescales

		Short‐term predictions^a^ about the direction and extent of trait change (1–50 generations)			Long‐term predictions about the direction and extent of trait change (e.g. 100+ generations)			Predictions if ancestral selection is re‐imposed
		Source of trait variation			Source of trait variation			
Change in selection	Original mode of trait expression	Environment^b^	Genes	Realised expression^c^	Environment	Genes	Realised expression	Realised expression
Relaxed (trait is neutral)	Constitutive	no change	no change	no change	no change	no change / ↓	no change / ↓	no change / ↓
	Context dependent	↓	no change	↓	↓	no change / ↓	↓ / ↓↓	no change / ↓
Reversed (trait is costly)	Constitutive	no change	no change / ↓	no change / ↓	no change	↓ / ↓↓	↓ / ↓↓	↓ / ↓↓
	Context dependent	↓	no change	↓	↓	no change / ↓	↓ / ↓↓	no change / ↓

^a^ ↓ indicates a small reduction in trait value; ↓↓ indicates a larger reduction in trait value.

^b^ Realised changes in expression are the combined effects of environmental and genetic sources of trait variation. After the ancestral selection regime is restored, only genetic sources of trait reduction will generally remain.

^c^ Plastic (environmental) changes involve behavioural flexibility or context dependence and are thus readily reversed if ancestral selection pressures are re‐imposed, whereas genetic changes in trait value will persist until selection acts to reverse them, and then only if heritable variation for the trait remains.

Phenotypic integration of behaviour is also likely to constrain its evolutionary reduction *via* vestigialisation, and one way in which behaviours may be strongly integrated with other adaptive behaviours or physiological processes is through pleiotropic constraint. Tierney (1996) discusses an example of behaviours strongly integrated by shared reliance on central pattern generators in Crustacea. Ecologically and physiologically distinct behaviours of ‘chewing’ by the gastric mill and swallowing are strongly integrated, relying on the same neuronal architecture. If selection against one form of chewing behaviour resulted in the loss or reduction of this behaviour, it would likely disrupt not only other forms of chewing behaviour regulated by the same neurons, but also completely distinct swallowing behaviours. Thus, behaviours which are underpinned by specific neural architecture are likely to be more evolutionarily responsive compared with behaviours that share neural architecture with other behavioural traits, especially if they serve distinct functions. Similarly, expression of non‐adaptive anti‐predator defence behaviours might persist in the absence of the associated predator, if they are integrated within a broader ‘vigilance’ behavioural syndrome which is maintained by other forms of predator threat (Blumstein, 2006).

While intuitive, it is not the case that context‐dependent behaviours are necessarily shielded from relaxed or reversed selection, and subsequent evolutionary reduction. A study of water fleas (*Daphnia magnia*) provides an instructive example (Cousyn *et al*., 2001). Water fleas resurrected from dormant propagules deposited during periods of high and low exposure to fish predation demonstrated rapid loss of plastic chemical‐induced predator‐avoidance behaviour. The majority (6 of 10) of clonal populations derived from sediment deposited during a period of high predation show plastic and putatively adaptive changes in behavioural phototaxis after exposure to chemical signals indicative of the presence of fish predators. By contrast, there was little or no evidence of a similar plastic response to the chemical signal among clonal populations derived from periods of low predation *ca*. 3 years before (0 of 10 showing predator‐mediated phototaxis) and 10 years after (1 of 10 showing predator‐mediated phototaxis) the temporary period of high predation. These results indicate that even context‐dependent behaviours can rapidly decay under relaxed (or reversed, if the chemical receptivity is costly to maintain) selection acting upon reaction norms. There was, however, evidence that the plastic response remained in the population after 10 years of relaxed selection, albeit at a lower frequency.

### (2) Timescale and genetic architecture

Timescale, or more specifically the number of generations that have elapsed since a change in selection, is likely to be an important factor affecting the persistence of vestigial behaviours and the likelihood of their evolutionary reduction. Most examples we have discussed appear consistent with the expectation that behaviours evolving under relaxed selection, such as those associated with behavioural responses to an absent predator, will persist over long evolutionary timescales (Coss, 1999; Lahti, 2006; Peer *et al*., 2011). By contrast, examples of behaviours that persist under reversed selection (i.e. which are deleterious or harmful) tend to have been characterised over relatively few (<100) generations (Magurran *et al*., 1995; Rayner *et al*., 2020). This persistent expression of harmful or costly behaviours is nevertheless notable given the comparatively rapid contemporaneous loss of associated morphological traits.

If non‐adaptive behavioural traits covary with other behavioural traits which retain adaptive value, then selection against them will be considerably weakened. Loss of behavioural traits under relaxed or reversed selection may also be hindered if genetic variation was purged by ancestral selection. Vestigial traits were, by definition, once adaptive so might have been subject to stabilising or directional selection which would diminish underlying genetic variation. This reduction in genetic variation will be particularly likely in cases where behaviours were previously strongly fitness associated. For example, although persistent singing behaviour is maladaptive in silent Hawaiian populations of the cricket *T. oceanicus*, it strongly influences male mating success in populations that harbour singing males (Tanner, Swanger & Zuk, 2019). This may explain in part the apparent widespread retention of anti‐predator behaviours under relaxed selection, as these traits are likely to have been historically subject to strong selection. Similarly, in cases of small effective population size, for example if a population has been exposed to genetic bottlenecks, vestigial behaviours might be retained due to the loss of associated genetic variation by genetic drift (Li *et al*., 2014).

## V. EVOLUTIONARY IMPLICATIONS OF VESTIGIAL BEHAVIOURS

### (1) Vestigial behaviours as an overlooked source of phenotypic variation

An outstanding question is whether vestigial behaviours have evolutionary consequences above and beyond those that might be expected from behaviour in general (Duckworth, 2009; Zuk *et al*., 2014; Bailey, Marie‐Orleach & Moore, 2018), or those of vestigial morphological or physiological traits. Behaviours which no longer serve their original function, which have been reduced in their expression to some intermediate state, or which are rarely expressed but for which the necessary genetic and physiological architecture remains, all have the potential to contribute to evolutionary dynamics because they represent phenotypic variation upon which future selection may act (Carson, 1978; West‐Eberhard, 2003; Tinghitella *et al*., 2018; Bailey *et al*., 2019). The evolutionary consequences therefore depend on whether the phenotypic variability of vestigial behaviour, or properties of its underlying genetics, contribute to distinctive evolutionary outcomes.

Behaviour has been commonly suggested to take an early lead in responding to changes in selection, due to its flexibility and potential to contribute to genetic evolution *via* phenotypic and genetic accommodation (Mayr, 1960; West‐Eberhard, 1989, 2003, 2005). Even if the behaviour is unexpressed, associated neural and genetic architectures might remain for many generations after the behaviour that together they produce is effectively lost. As an example, field crickets (*Gryllus ovisopsis*) which do not produce the type of long‐range calling song typically observed in related species to attract mates, nevertheless retain the ability to produce wing motor patterns associated with calling song (Gray *et al*., 2018). Despite not expressing calling behaviour under natural circumstances, singing behaviour can be experimentally induced by administering exogenous neurotransmitters to activate neuronal circuits underlying the appropriate wing movements (Gray *et al*., 2018). Similarly, sedentary grasshoppers (*Phymateus morbillosus*) which due to their large size exhibit marginal or no flight ability, can still express the necessary wing movement patterns (Kutsch *et al*., 2002). These examples underscore how the intrinsic relationship between behaviour and neural physiology can allow behaviour to persist in a functionless, reduced, or even unexpressed but ‘potential’ state, providing a substrate upon which future selection might act.

The ultimate evolutionary consequences of vestigial behaviours are likely to depend on the extent of such pleiotropic constraints acting upon them. Strong phenotypic integration caused by pleiotropy could, as well as contributing to their non‐adaptive persistence, also constrain the degree to which vestigial behaviours respond to selection favouring new functions. For example, in insects such as *Drosophila melanogaster* and birds such as dark‐eyed juncos (*Junco hyemalis*), expression of signalling molecules such as juvenile hormone and testosterone, respectively, affects an extremely wide range of functions, from behaviour to life‐history transitions, to the development of morphological variations (Flatt, Tu & Tatar, 2005; McGlothlin & Ketterson, 2008). However, it has been argued that pleiotropic integration can be highly modular; that is, pleiotropic constraint may be strong yet restricted to a relatively narrow range of functionally related traits (Wagner & Altenberg, 1996; Wagner & Zhang, 2011), and secondly that phenotypic integration might in some cases facilitate rather than impede diversification *via* adaptive radiations, as was recently found in Darwin's finches and Hawaiian honeycreepers (Navalón *et al*., 2020). Vestigial behaviours might represent a potentially rich source of phenotypic variation upon which new forces of selection may act, due to lack of recent stabilising or directional selection, and the context‐ and state‐dependent properties of behaviour. Whether vestigial behaviours contribute unusually to diversification and adaptive innovation may depend on underlying pleiotropic constraints, but even if they are integrated with other traits, it is reasonable to predict that their existence could create favourable conditions for onward evolutionary adaptation, diversification, and innovation.

### (2) Vestigial behaviours and preadaptation, diversification, and innovation

“A structure is said to be preadapted for a new function if its present form which enables it to discharge its original function also enables it to assume the new function whenever need for this function arises.”

Bock (1959).

All novel adaptations logically proceed from pre‐existing traits. For this simple reason, pointed concerns have historically been raised about the usefulness of concepts such as ‘preadaptation’, ‘preaptation’, ‘exaptation’, and ‘co‐option’ (Bock, 1959; Gould & Vrba, 1982). Nevertheless, when an existing trait, including a vestigial trait, has the potential to acquire a new adaptive advantage not due to evolutionary changes in the trait itself but rather a change in selective pressure which favours it for a function other than that for which it was previously selected, it may be considered to confer elevated evolutionary potential along a particular phenotypic trajectory. That is, such a trait's existence within a population means that the likelihood of particular evolutionary changes is, on average, enhanced compared with a population not containing such preadaptations. Vestigial behaviours may therefore act as evolutionary capacitors, predisposing the evolution of innovations of a particular form and function, such as has been shown for the repeated evolution of snapping claws in alpheid shrimps (Anker *et al*., 2006). A more expansive view is that vestigial behaviours actually confer greater evolutionary potential precisely because they are released from functional constraints. A non‐behavioural example occurs in treehoppers in the insect family Membracidae, in which an additional wing‐like appendage is expressed in the first thoracic segment in contrast to virtually all other insect taxa. This wing‐like outgrowth is not under selective pressure for flight, since flight wings already exist on other thoracic segments, and as a result it has evolved into an extraordinary array of elaborated ‘helmets’ across different species within the group (Prudhomme *et al*., 2011).

We note two ways in which the distinct evolutionary potential of vestigial behaviours as preadaptations might be realised. One is for a change in selection pressure to favour a previously reduced function or functionless behaviour, in which case it increases organismal fitness and thus a population's proximity to a fitness optimum. This process supposes that one vestigial behaviour leads to the evolution of a trait with a new function when selection changes; a one‐for‐one evolutionary replacement. However the other mechanism is for a pre‐existing vestigial behaviour to facilitate phenotypic diversification, such that many evolutionary novelties or even new species are produced. For example, it has been suggested that removal of selection on song function in domesticated Bengalese finches (*Lonchura striata*) has resulted in a more complicated and inter‐individually variable song repertoire, which is transmitted by social learning (Deacon, 2010). Other signal–receiver systems have been found to exhibit similar dynamics. An anti‐predator startle response in lebinthine crickets from Papua New Guinea appears to have evolved into a complex duetting behaviour through sensory exploitation (ter Hofstede *et al*., 2015). When the ultrasonic signals of bats are absent, the male crickets produce high‐frequency ultrasonic calls which release female vibrational signals. In closely related species, however, individuals display an anti‐predator startle response instead of a vibrational signal; thus the startle response appears to have evolved into a novel mate recognition system as a result of selection on a behaviour with an ancestral function in avoiding predators.

In other instances, when morphological trait reduction exposes underlying phenotypic variation, vestigial behaviours which were previously involved in expressing the morphological trait might expose that variation to selection. There is evidence in support of this in the *Teleogryllus oceanicus* field cricket system mentioned above, in which morphological trait loss has eliminated a sexual signal but left behind vestigial singing behaviour in the form of wing movements (Schneider *et al*., 2018; Rayner *et al*., 2020). In this system, it has been shown that unexpressed signal properties of the disrupted wing membranes are both more variable and strikingly different from the ancestral ‘wild‐type’ cricket song (Bailey *et al*., 2019). Emerging evidence suggests that re‐evolution of new signals in the wild may be underway (Tinghitella *et al*., 2018), supporting this model of signal diversification through the persistence and subsequent functional enhancement of vestigial behaviours. By an analogous process, highly variable vestigial signalling behaviours could provide a phenotypic substrate for the evolution of redundant signals or signal components.

## VI. FUTURE RESEARCH

### (1) Identifying vestigial behaviours

Examples of apparently vestigial behaviours abound, but studies are rarely targeted towards identifying and explaining these behaviours themselves. An important first consideration is how vestigial behavioural traits are to be identified and whether this needs to differ from the way other vestigial traits are identified, with the key difficulties relating to determining directionality of the change in selection and specifically identifying the trait's ancestral state and its degree, if any, of diminution. In the most straightforward cases, this will be determined by pre‐existing knowledge of the study population(s), as in longitudinal research programmes during which changes in selection pressure are observed, experimentally manipulated, or documented (Reznick *et al*., 1990; Wund *et al*., 2015). Alternatively, historical selection pressures might be readily inferred after an observed change in a population's environment, for example following colonisation of a new habitat in which certain ancestral selection pressures are absent: such as the absence of a key predator in the species' ancestral range (Lahti, 2006), or colonisation of underground caves rendering eyesight non‐adaptive (Jeffery, 2005). If such a change has taken place outside of an observable period, for example if it occurred over a geological timescale, then the direction of change should be confirmed by genetic analysis (i.e. derived and ancestral population designations should be confirmed). Phylogenetic analysis may also provide a useful framework to identify potentially vestigial forms of behaviour, as well as identifying behavioural variation associated with phylogenetic signal more generally, which recent research has indicated might account for a substantial proportion of observed variation (Dalos *et al*., 2021; but see White, Pascall & Wilson, 2020). Selection pressures at the relevant time points should also where possible be corroborated – for example by historical record, metagenomics or geological/fossil records (Cousyn *et al*., 2001) – rather than by assuming that contemporary differences in selection regime are representative of long‐term trends. Even when differences in selection pressures are identified, researchers should be wary of treating behaviours as strictly non‐adaptive or costly without further evidence, although fitness effects may be difficult to assess particularly for context‐dependent behaviours that remain largely unexpressed in the absence of associated cues.

A feature of vestigial behaviours which renders them distinct from many other forms of vestigial trait is that they are often highly variable in expression at the individual level. Thus, a key goal is to reliably quantify expression of the behaviour in question, for example by taking repeat measurements and obtaining a sample size that is sufficient to be considered representative of the population, rather than relying on simplistic measures such as observed presence/absence. Without quantitative data it is often not clear whether vestigial behaviours are reduced in their expression or remain unattenuated. Care must be taken to define precisely what constitutes diminution: unlike morphological traits, extant behaviour may be expressed with perfect fidelity to ancestral behaviour, but simply less often, or with higher thresholds of induction. Such a reduction is qualitatively different to other forms of reduction that involve dropping elements of a movement pattern or a signalling repertoire.

A further issue to consider is the conditions under which behaviours are measured in laboratory experiments, particularly when comparing populations evolving under different selection regimes. Ideally, expression would be quantified *in situ* for each of the populations, as well as under controlled laboratory conditions. Thus, both realised expression and additive genetic differences in expression level are assayed, illuminating whether any observed differences in expression appear primarily due to behavioural flexibility or plasticity (e.g. if expression of the behaviour is experience based), or to evolved genetic differences. To assay the latter reliably, populations should where possible be bred under laboratory conditions for at least one generation prior to experiments to avoid experiential and parental effects. However, in many cases it is difficult or unfeasible to quantify expression of the behaviour in the wild, particularly if the behaviour is highly context dependent, or if expression relies on morphological traits that have been lost or strongly reduced. In these cases, comparisons will necessarily proceed under laboratory conditions, in which case it is important to be cognisant of and account for differences in selection regime between populations above and beyond the selection pressure of interest. For example, populations may have genetically adapted to differences in thermal regime, in which case expression of the behaviour of interest may scale differently with temperature in the two populations. If the behaviour is only assayed under one condition, the results may be confounded by this unaccounted‐for variation. Where such differences are recognised, respective expression levels will be most reliable when quantified under both sets of conditions.

### (2) Testing the causes and consequences of vestigial behaviours

Many examples of vestigial behaviours have been identified through applied research, such as conservation of threatened species, or by happenstance. More rarely have researchers set out to study the fate of behaviours under relaxed selection, or selection reversal, yet this is a worthy goal in itself. Behaviour is often touted to be particularly important in adapting to rapid environmental change of the sort imposed by anthropogenic activity, but it is important also to appreciate the constraints that might impede such adaptation. Vestigial behaviour research could also help predict the route adaptation may take, if vestigial behaviours predispose populations to certain evolutionary trajectories, or otherwise represent a form of evolutionary contingency.

Missing in the studies we have discussed is an in‐depth genetic analysis of vestigial behaviours, which could take the form of genomic studies or quantitative genetic experiments that identify and quantify constraints, such as those imposed by patterns of covariance among suites of behavioural traits (Dochtermann & Dingemanse, 2013; Royauté *et al*., 2020). Thus, for most of the examples discussed, proposed explanations for the persistence of the behavioural trait under relaxed or reversed selection lack empirical evidence. In cases where the genetic architecture of behavioural expression can be or has been characterised, for example by quantitative trait locus (QTL) mapping, genomic analyses could test whether regions of the genome that are associated with expression of a vestigial behaviour show signatures of relaxed selection, or whether they appear to remain under selection, for example due to pleiotropic constraints. More generally, it is important to understand to what extent behavioural reduction or loss, where it is observed, is underpinned by genetic evolution *versus* plastic, experience‐based means. For example, if behavioural trait reduction occurs largely through genetic changes, then efforts to maintain predator‐defence behaviours in captivity by simulating predator presence (Greggor *et al*., 2021) may fail as there is no associated selection pressure. This could suggest the maintenance of low‐level predator exposure may be a more suitable approach (West *et al*., 2018), potentially even if it is not the same species the population is exposed to in the wild (Blumstein, Daniel & Springett, 2004).

Future research might also involve experimental evolution studies, which have yielded considerable insight into the evolutionary dynamics of trait loss under relaxed selection (Card *et al*., 2019). However, when such experimental studies have addressed the relaxation of selection on behavioural traits, they have often done so by first subjecting the trait to several generations of strong bidirectional selection, before ceasing the selection regime (Dobzhansky & Spassky, 1969; Matsumura & Miyatake, 2018; Souto‐Maior, Serrano Negron & Harbison, 2020). Inferences drawn from these studies might therefore be limited by the purging of genetic variation by directional selection regimes, and by focussing on extreme traits which likely confer strong fitness costs. Notable exceptions include the evolutionary loss of mating behaviours in experimental parthenogenetic lines of *Drosophila* (Carson *et al*., 1982), revealing heterogeneity in response to selection across replicates. An intriguing experimental design may involve relaxing selection on a given behavioural trait, or suite of behavioural traits, and investigating how the response to relaxed selection affects evolutionary dynamics if novel or ancestral selection pressures are imposed.

Another interesting avenue for research will be to investigate whether social learning can and does contribute to the retention of non‐adaptive behaviours. As for behavioural variation more generally, forms of social learning are widely appreciated as a potential source of adaptation to changing conditions, particularly those imposed by humans (Brakes *et al*., 2021). Nevertheless, proliferation of behaviours by social learning can also lead to the spread of non‐adaptive forms of behaviour (Curio, 1993; Deacon, 2010), as long as the behaviour does not impose very strong fitness costs. For instance, a population of chimpanzees (*Pan troglodytes*) was observed to exhibit socially learned, apparently non‐adaptive behaviour involving placing straw‐like blades of grass into their ears (van Leeuwen, Cronin & Haun, 2014). In this case it is doubtful the behaviour ever conferred an adaptive benefit, but it is intriguing to consider whether traits which originally evolved under selection could be similarly retained by social transmission once the trait itself loses an adaptive benefit.

## VII. CONCLUSIONS

1. It is now widely appreciated that, while much of the behavioural variation observed in animals is adaptively shaped, a substantial portion of this variation is not currently adaptive. Our review highlights the importance of such variation through examination of cases in which behaviours remain expressed or capable of being expressed after the selective pressures that favoured their maintenance are relaxed or eliminated.
2. Vestigial behavioural traits appear to persist at least as commonly under relaxed or reversed forms of selection as do morphological or physiological traits, but are less widely appreciated. In fact, the context‐specific expression and other features of behavioural traits appear particularly to predispose them to longer‐term persistence under relaxed selection. This pattern of non‐adaptive behavioural persistence has come to be relatively well appreciated among traits involved in predator–prey interactions but is by no means unique to this ecological context and so warrants broader recognition and research.
3. The circumstances that favour the maintenance of vestigial behaviours nevertheless remain unclear. Behaviours that are under relaxed selection due to context‐specific expression can persist for many generations. However, even costly and frequently expressed behaviours may also persist over many generations if they exhibit features such as low heritability or are subject to strong pleiotropic constraints.
4. Of particular interest is the role that these vestigial behaviours might play in downstream evolutionary dynamics. Does the maintenance of non‐adaptive variation favour diversification, or promote future adaptation? Vestigial behaviours frequently regain adaptive value when ancestral conditions are restored but might also be co‐opted for novel functions or during diversification.
5. Study of vestigial behaviours will benefit from analyses of underlying genetic changes. Few studies have examined genetic variation associated with behavioural traits experiencing relaxed or reversed selection. This is an important gap in knowledge. For example, if behavioural trait reduction is generally underpinned by genetic changes under relaxed selection, efforts to maintain behaviours over long periods in captivity, wildlife refuges or laboratory conditions may fail in the absence of associated selection pressures.
